# New insights into CAR T-cell hematological toxicities: manifestations, mechanisms, and effective management strategies

**DOI:** 10.1186/s40164-024-00573-9

**Published:** 2024-11-09

**Authors:** Yuanyuan Yang, Hongwei Peng, Jianxiang Wang, Fei Li

**Affiliations:** 1 Jiangxi Provincial Key Laboratory of Hematological Diseases, Department of Hematology, The First Affiliated Hospital, Jiangxi Medical College, Nanchang University, Nanchang, Jiangxi China; 2 Department of Pharmacy, The First Affiliated Hospital of Nanchang University, Nanchang, Jiangxi China; 3 State Key Laboratory of Experimental Hematology, Haihe Laboratory of Cell Ecosystem, Institute of Hematology and Blood Diseases Hospital, National Clinical Research Center for Blood Diseases, Chinese Academy of Medical Sciences and Peking Union Medical College, Tianjin, China; 4 Jiangxi Clinical Research Center for Hematologic Disease, Nanchang, Jiangxi China; 5 Institute of Lymphoma and Myeloma, Nanchang University, Nanchang, Jiangxi China

**Keywords:** Chimeric antigen receptor (CAR), CAR T-cell therapy, Hematological toxicity, Cytopenias, Coagulopathy, HLH, B-cell aplasia

## Abstract

Chimeric antigen receptor (CAR) T-cell therapy represents a highly efficacious treatment modality demonstrated to enhance outcomes in patients afflicted with malignancies, particularly those enduring relapsed or refractory hematological malignancies. However, the escalating adoption of CAR T-cell therapy has unveiled several life-threatening toxicities, notably cytokine release syndrome (CRS), immune effector cell-associated neurotoxicity syndrome (ICANS), infections, and hematological toxicities (HTs), thereby hindering the broad implementation of CAR T-cell therapy. HTs encompass a spectrum of adverse effects, including cytopenias, hemophagocytic lymphohistiocytosis (HLH), coagulopathies, and B-cell aplasia. While our comprehension of the underlying mechanisms governing CRS and ICANS is advancing, the intricate pathophysiology of HTs remains inadequately elucidated. Such knowledge gaps may precipitate suboptimal therapeutic decisions, potentially culminating in substantial medical resource depletion and detriment to patients’ quality of life. In this comprehensive review, based on recent updated findings, we delineate various mechanisms contributing to HTs subsequent to CAR T-cell therapy, explicate manifestations of HTs, and proffer strategic interventions to mitigate this relevant clinical challenge.

## Background

Chimeric antigen receptor (CAR) T-cell therapy has emerged as a promising therapeutic strategy. This therapeutic approach has not only demonstrated significant efficacy but has also catalyzed a paradigm shift in the management of patients afflicted with relapsed/refractory (R/R) hematological malignancies [1].To date, six CAR T-cell therapeutic products have garnered approval from the U.S. Food and Drug Administration (FDA) for the treatment of R/R B-cell acute lymphoblastic leukemia (B-ALL), diffuse large B-cell lymphoma (DLBCL), follicular lymphoma (FL), mantle cell lymphoma (MCL), chronic lymphocytic leukemia/small lymphocytic lymphoma (CLL/SLL) and multiple myeloma (MM). Additionally, five products have been approved by the National Medical Products Administration (NMPA) in China for the treatment of R/R B-ALL, large B cell lymphoma (LBCL), FL, MCL and MM, as illustrated in Table 1 [2–8].

While numerous clinical trials investigating CAR T-cell therapy have yielded unprecedented positive outcomes, they are often accompanied by a distinctive array of toxicities that can pose life-threatening risks and, at times, constrain the widespread adoption of CAR T-cell therapy [9–11]. Notably, toxicities such as cytokine release syndrome (CRS) and immune effector cell-associated neurotoxicity syndrome (ICANS), whose pathophysiology is well-documented and elucidated in the scientific literature, have prompted the development of increasingly effective strategies for toxicity management [10, 12–14]. Hematological toxicities (HTs) represent a frequently encountered adverse event, including cytopenias, hemophagocytic lymphohistiocytosis (HLH), coagulopathies, and B-cell aplasia, with an incidence exceeding 90% [9, 11, 15–18]. Their association with unfavorable outcomes has garnered considerable attention, while the pathophysiology and managements of HTs remains insufficiently elucidated.

The aim of this review is to provide a comprehensive overview of the various HTs that triggered after CAR T-cell therapy, including cytopenias, HLH, coagulopathies, and B-cell aplasia. This overview will include an examination of the underlying mechanisms, risk factors, clinical manifestations, management strategies and disease prognosis associated with these HTs.

**Table 1:** FDA and NMPA approved CAR T-cell therapies

Organization	Production	Target	Year	Indication
FDA	Tisagenlecleucel (Kymriah)	CD19	2017	R/R B-ALL
			2018	R/R DLBCL
	Axicabtagene ciloleucel (Yescarta)	CD19	2017	R/R LBCL
			2021	R/R FL
	Brexucabtagene autoleucal (Tecartus)	CD19	2020	R/R MCL
			2021	R/R B-ALL
	Lisocabtagene maraleucel (Breyanzi)	CD19	2024	R/R FL
			2024	R/R MCL
			2024	R/R CLL/SLL
	Idecabtagene vicleucel (Abecma)	BCMA	2021	R/R MM
	Ciltacabtagene autoleucel (Carvykti)	BCMA	2022	R/R MM
NMPA	Axicabtagene ciloleucel (Yescarta)	CD19	2021	R/R LBCL
	Relmacabtagene autoleucel	CD19	2021	R/R LBCL
			2022	R/R FL
			2024	R/R MCL
	Inaticabtagene autoleucel	CD19	2023	R/R B-ALL
	Equecabtagene autoleucel	BCMA	2023	R/R MM
	Zevorcabtagene autoleucel	BCMA	2024	R/R MM

Abbreviations FDA, food and drug administration; NMPA, national medical products administration; CAR, Chimeric Antigen Receptor; CD19, Cluster of Differentiation 19; R/R: relapsed/refractory; B-ALL, acute B lymphoblastic leukemia; DLBCL, diffuse large B cell lymphoma; FL, follicular lymphoma; MCL, mantle cell lymphoma; MM, multiple myeloma; LBCL, large B cell lymphoma; CLL/SLL: Chronic Lymphocytic Leukemia/Small Lymphocytic Lymphoma; BCMA, B cell maturation antigen

## Cytopenias

**Fig. 1:**
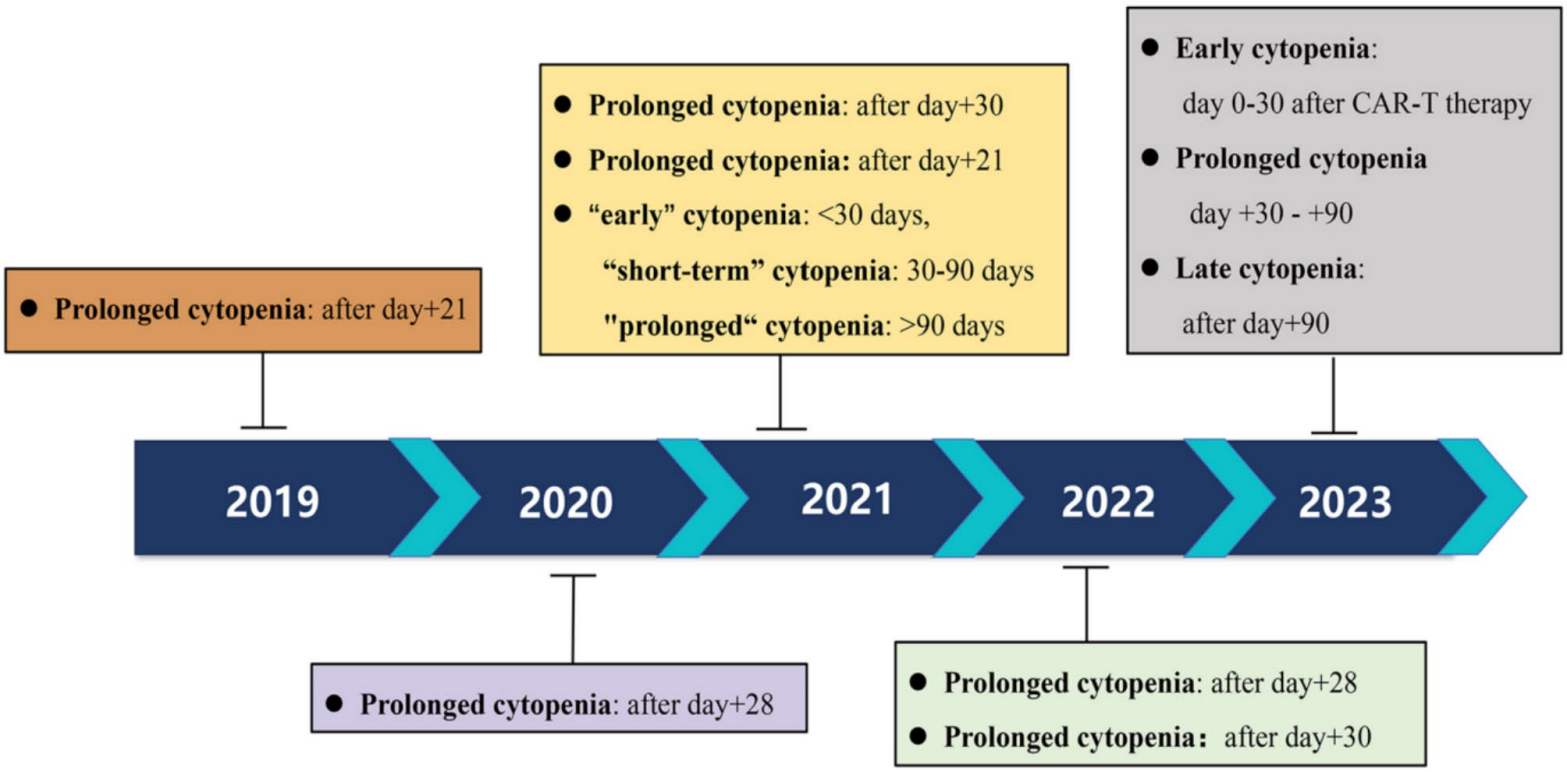
Temporal definition of prolonged cytopenia in different studies from 2019 to 2023 [15, 17, 19, 20, 28–32]

Cytopenias subsequent to CAR T-cell infusion are typically categorized into three distinct groups based on their onset and duration. These are early cytopenias (0–30 days), long-term cytopenias (30–90 days) and late cytopenias (after 90 days). All three categories are accompanied by grade 3/4 cytopenias (according to the National Cancer Institute’s Common Terminology Criteria for Adverse Events) [13]. Previously, the absence of clear guidelines and consensus regarding the staging of cytopenia following CAR T-cell therapy has resulted in discrepancies across various studies. Consequently, research findings, including the reported incidence of cytopenia and associated risk factors, have exhibited inconsistencies (Table 2; Fig. 1). Cytopenias subsequent to CAR T-cell therapy have recently been classified as immune effector cell-associated hematological toxicity (ICAHT) by the European Hematology Association (EHA) and the European Society for Blood and Marrow Transplantation (EBMT) [19]. ICAHT has been documented to manifest with a notably high incidence in both real-world settings and clinical trials following CAR T-cell therapy (Table 2), leading to prolonged transfusion requirements and heightened risks of infection, bleeding, and bruising [15, 19–21]. Previous investigations have underscored that neutropenia-associated infections represent the predominant cause of non-relapse mortality among CAR T-cell recipients, surpassing both CRS and ICANS [22].

**Table 2:** The incidence of cytopenias following CAR T-cell therapy

Study	Trail ID	The threshold of prolonged/late cytopenia	Malignancy	*N*	Target	Costimulatory domain	Toxicity scale	Disease entity *n* (%)	Early cytopenias *n* (%)	Prolonged/late cytopenias *n* (%)
Neelapu, et al. [23]	NCT02348216	NA	R/R LBL	101	CD19	CD28	CTCAE v4.03	Neutropenia 48 (84) Thrombocytopenia 59 (58) Anemia 73 (66)	NA	NA
Fried, et al. [15]	NCT#02772198	21d	R/R B-ALL R/R B-NHL	35	CD19	CD28	CTCAE v4.03	NA	Neutropenia 11 (31) Thrombocytopenia 6 (17) Anemia 13 (37)	Neutropenia 22 (63) Thrombocytopenia 22 (63) Anemia 5 (14)
Locke, et al. [24]	NCT02348216	NA	refractory LBCL	101	CD19	CD28	CTCAE v4.03	Neutropenia 48 (44) Thrombocytopenia 38 (35) Anemia 73 (68)	NA	Neutropenia 12 (11) Thrombocytopenia 6 (7) Anemia 3 (3)
Rejeski, et al. [20]	NA	21d	R/R LBCL	259	CD19	4-1BB CD28z	CTCAE v4.03	NA	Neutropenia 151 (36)	Neutropenia 151 (64)
Schuster, et al. [25]	NCT02445248	28d	R/R LBCL	115	CD19	4-1BB	CTCAE v4.03	Neutropenia 40 (35) Thrombocytopenia 39 (34) Anemia 55 (48)	Neutropenia 1 (2.5) Thrombocytopenia 6 (15.3) Anemia 3 (5)	Cytopenias 39 (34)
Li, et al. [17]	ChiCTR-OIC-17,011,272	28d	R/R MM	54	BCMA/CD19	4-1BB	CTCAE v5.0	Neutropenia 48 (89) Thrombocytopenia 29 (53) Anemia 26 (48)	NA	Cytopenias 28 (52)
Wang, et al. [26]	ChiCTR1800017404	30d	R/R MM	93	BCMA	4-1BB	CTCAE v5.0	NA	NA	Neutropenia 36 (38.71) Thrombocytopenia 55 (59.14) Anemia 21 (22.58)
Jess, et al. [27]	NCT02315612	NA	R/R B-ALL R/R DLBCL	62	CD22	4-1BB	CTCAE v4.0	Neutropenia 51 (81) Thrombocytopenia 19 (30)	NA	NA
Rejeski, et al. [19]	NA	30d	R/R MM R/R LBCL R/R MCL	549	BCMA CD19	4-1BB CD28z	ICATH	Severe Thrombocytopenia 229 (57) Severe Anemia 240 (60)	NA	NA

(A) The threshold of prolonged/ late cytopenias: the duration of sustained grade 3 or higher cytopenias from the time of CAR T-cell infusion, which exceeds 28–30 days; N means the total number of patients; (B) abbreviations: R/R: relapsed/refractory; LBCL, large B cell lymphoma; B-ALL, acute B lymphoblastic leukemia; B-NHL, B-cell non-Hodgkin lymphoma; MM, multiple myeloma; DLBCL, diffuse large B cell lymphoma; MCL, mantle cell lymphoma. BCMA, B cell maturation antigen; NA, not available; CTCAE, Common Terminology Criteria for Adverse Events; ID, identification

### Risk factors for cytopenias

#### Baseline characteristics

Prior to lymphodepletion (LD) chemotherapy, several risk factors have been identified that correlate with the occurrence of cytopenias subsequent to CAR T-cell therapy. A history of more than three prior lines of chemotherapy is associated with an increased risk of late cytopenias [33]. Moreover, regarding disease characteristics, B-ALL appears to confer a higher risk compared to B-cell lymphoma, although this assertion remains subject to debate [16, 24]. Additionally, baseline cytopenias, elevated disease burden, a history of allogeneic hematopoietic stem cell transplantation (allo-HSCT) within the preceding year, and elevated levels of serum/plasma inflammatory markers, such as ferritin and C-reactive protein (CRP), have been positively correlated with the development of cytopenias subsequent to CAR T-cell therapy [16, 34–38]. The CAR-HEMATOTOX model that has been validated in MM and B cell lymphoma incorporates baseline markers associated with inflammation and hematopoietic reserve to estimate a patient’s risk of experiencing early and late cytopenias with high sensitivity [19]. The factors contributing to the development of cytopenias subsequent to CAR T-cell therapy primarily encompass those delineated in Table 3.

**Table 3:** Risk factors for cytopenias post-CAR T-cell therapy

Risk factors	Early cytopenias	Prolonged cytopenias	Late cytopenias
baseline characteristics	Infection [39] prior radiotherapy [40] prior HSCT [35, 41] higher baseline ferritin & CRP [40]	prior-HSCT<1 year [41] > 3 prior therapies [42] higher baseline ferritin & CRP [40]	Cytopenias (ANC, Hb, PLT) [20, 41] higher baseline ferritin & CRP [40] > 3 prior therapies [42] Prior-CHIP [43]
disease related	baseline tumor burden [20]	baseline tumor burden [20] secondary malignancy [41]	baseline tumor burden [20] secondary malignancy (MDS) [41]
CAR T-cell- related	Infection [41] IEC-HS [40, 41] LD [41] CRS [22, 40, 44, 45] ICANS [22, 40, 44, 45] CAR constructs [16]	Infection [41] IEC-HS [41] higher maximum D-dimer [46] CAR constructs (4-1BB-based CAR-T cells) [15] expansion of CAR-T cells [26, 47] higher maximum IFN-γ, IL-6, IL-10 [17]	CRS grade ≥ 4 [48] ICANS grade ≥ 3 [16] bone marrow destruction [49] CAR constructs [15] expansion of CAR-T cells [47] Low CD4^+^/CD8^+^T cell ratio of CAR-T cells [26] severe hematologic toxicity after LD [41]

Abbreviations: HSCT, hematopoietic stem cell transplantation; CRP, C-reactive protein; ANC, absolute neutrophil count; HB, hemoglobin; PLT, platelet; CHIP, clonal hematopoiesis of indeterminate potential; MDS, myelodysplastic syndrome; IEC-HS, immune effector cell-associated HLH-like syndrome; LD, lymphodepletion; ICANS, immune effector cell-associated neurotoxicity syndrome; IFN-γ, interferon γ

#### Conditioning chemotherapy

Conditioning chemotherapy, a crucial step performed prior to CAR T-cell infusion, typically involving the administration of fludarabine/cyclophosphamide (FC), aimed at facilitating CAR T-cell engraftment [50]. Early cytopenias are anticipated owing to the myelotoxic effects of FC [15]. Recently, Ghilardi et al. have demonstrated that the utilization of bendamustine prior to tisagenlecleucel treatment yields comparable efficacy alongside a reduced incidence of cytopenias in comparison to FC [51]. However, this explanation alone fails to fully elucidate the extent of long-term cytopenias following CAR T-cell administration, the precise mechanism underpinning this phenomenon remains elusive [15, 52].

#### Cytokine release syndrome (CRS)

CAR T-cell-induced inflammation constitutes a pivotal determinant in the development of HTs, numerous investigations have underscored the association between cytopenias and high-grade CRS [15, 20, 22, 46, 53]. Higher-grade CRS, ICANS, and immune effector cell-associated HLH-like syndrome (IEC-HS) are correlated with lower hematologic nadirs, delayed hematopoietic recovery, and heightened transfusion dependency [16, 41, 42, 53] (Table 3). The precise role of cytokines, including interferon (IFN) (class II cytokine), tumor necrosis factor (TNF) family, growth factors, IL-1 family, IL-17 family, chemokines, and hematopoietin receptor family (class I cytokine), in the genesis of HTs remains contentious, despite extensive examination in numerous clinical studies [15, 16, 46, 53, 54]. Elevated serum concentrations of IL-6, IFN-γ, and IL-10 have been identified as risk factors for the development of cytopenias [46, 53]. Conversely, higher serum levels of transforming growth factor-β1 (TGF-β1) and serum stromal cell-derived factor-1 (SDF-1) have been associated with enhanced recovery of blood counts [15, 53].

Additionally, IL-2 serves as a hematopoietic stimulant capable of potentially mitigating long-term cytopenias. Within the immune milieu, IL-2 facilitates regulatory T cell (Treg) survival and suppressive function while constraining CD8^+^ T-cell activation, thereby potentially curbing CRS progression and mitigating the severity of cytopenias [26].

Notwithstanding the strong correlation between CRS and cytopenias may persist even subsequent to CRS remission. We hypothesized that elevated cytokine levels (e.g. IL-6, IL-10, IFN-γ) may cause chronic inflammation of HSCs and then impede the hematopoietic system’s capacity to generate various lineages, thereby engendering a delayed hematopoietic recovery.

#### Characteristics of CAR T-cells

Despite the manufacturing process, structural composition, and origin species of the single-chain Fv (scFv) utilized in CAR T-cell engineering exert notable influences on the immune response and therapeutic efficacy, the precise impact of differences in the CAR constructs on cytopenias remains unclear [16, 55]. Luo et al. identified that CAR T-cells incorporating a CD28 costimulatory domain tends to exhibit higher incidences of thrombocytopenia and anemia in patients compared to those employing a 4-1BB costimulatory domain [56]. Nevertheless, in Jain’s study, the correlation between CAR structure and cytopenias was identified as statistically significant in univariate models but was no longer significant in multivariate analysis [16]. Currently, there is a paucity of studies examining the relationship between CAR structure and cytopenias. Moreover, the various subtypes of CAR T-cells have also been identified as a contributing factor to the prolonged/late cytopenias. The low CD4/CD8 ratio of CAR T-cells has been established as a risk factor for the prolonged cytopenias. Additionally, a longer duration of CAR-T cell expansion has been linked to an increased risk of long-term haematopenia in patients [26, 41, 47] (Table 3). To gain further insight, larger clinical studies are required.

### Mechanisms of cytopenias

#### Sustained inflammation-mediated cytopenias

**Fig. 2:**
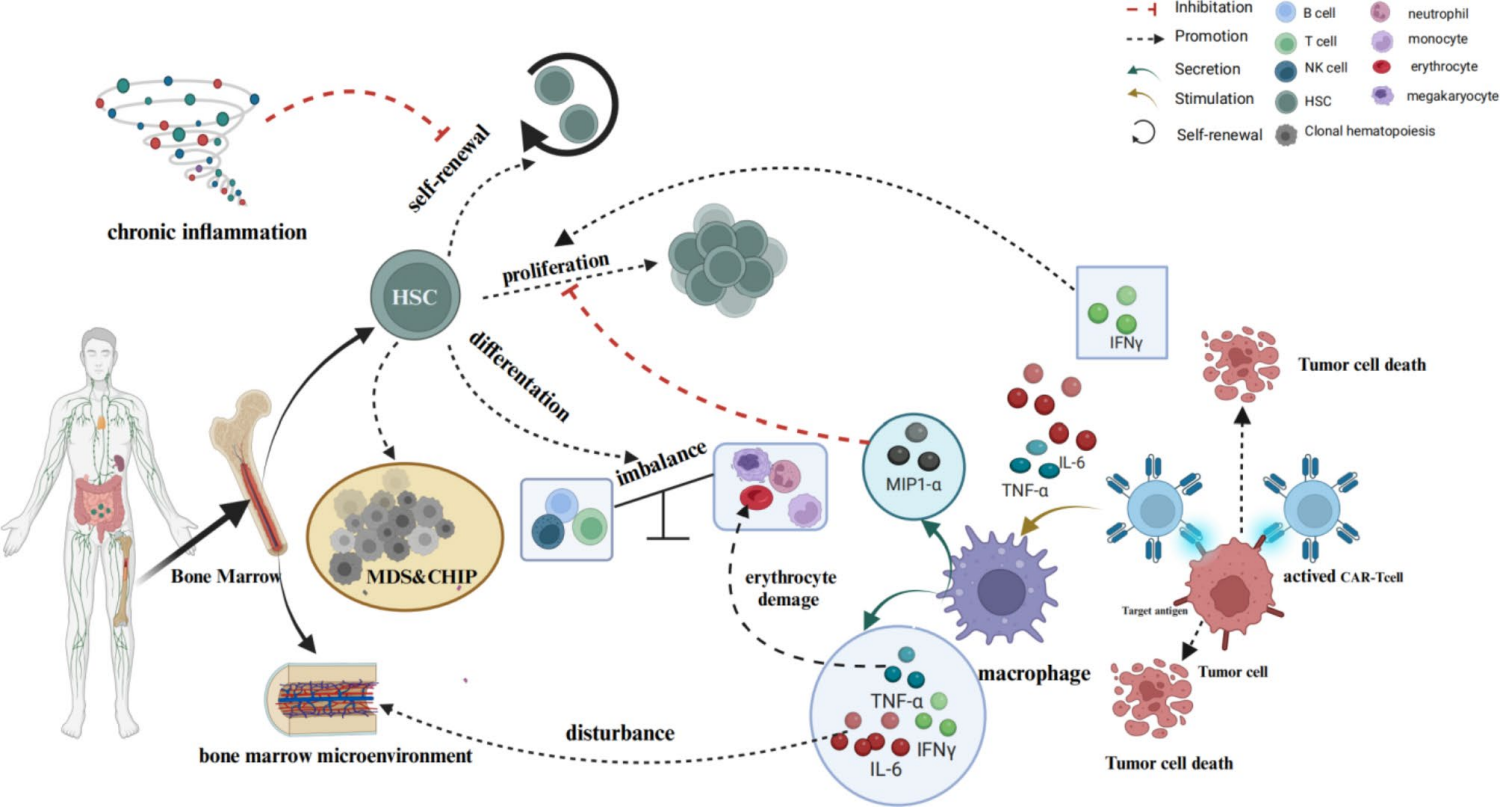
Potential mechanisms of cytopenias after CAR T-cell therapy. CAR T-cells become activated upon binding to tumor cells, triggering the release of inflammatory cytokines such as IL-6, TNFα, IFN-γ, among others. Concurrently, the demise of tumor cells results in the release of inflammatory soluble factors, including IL-6 and IL-18. IFN-γ, in particular, fosters the proliferation of HSCs. However, persistent stimulation from chronic inflammation leads to the excessive proliferation of HSCs, ultimately depleting their self-renewal capacity. Furthermore, activated macrophages release a diverse array of cytokines, including IL-6, TNFα, IFN-γ, and MIP1-α, which disrupt the BMM and perturb the balance of HSC differentiation into myeloid and lymphoid cells. Notably, MIP1-α has been observed to inhibit HSC proliferation. Additionally, CHIP and MDS have been linked to long-term cytopenias following CAR T-cell therapy. Abbreviations: CAR, chimeric antigen receptor; HSC, hematopoietic stem cell; IFN-γ, interferon-γ; IL, interleukin; TNF-α, tumor necrosis factor-alpha; MCP-1α, monocyte chemoattractant protein-1alpha; NK cell, natural killer cell

The prevailing body of research suggests that baseline hematopoietic reserve and inflammatory status are closely associated with the occurrence of prolonged cytopenias [16, 20]. Rejeski et al. have introduced the “CAR-HEMATOTOX score”, incorporating the inflammatory markers including ferritin and CRP, alongside blood counts, to prognosticate the risk of enduring cytopenias subsequent to CAR T-cell infusion [20]. Upon engagement with tumor cells, CAR T-cells undergo activation, releasing an array of inflammatory cytokines, including IFN-γ, IL-6, IL-10, TNF-α, and granulocyte macrophage-colony stimulating factor (GM-CSF). Concurrently, the lysis of tumor cells liberates additional inflammatory cytokines such as IL-18 and IL-6 [57]. In particular, the proinflammatory cytokine IL-6, pivotal in CRS, has been implicated in attenuating the function of erythroid progenitor cell populations [16, 53, 58]. While IFN-γ fosters HSC proliferation, sustained inflammatory stimulation can precipitate HSC over-proliferation, ultimately exhausting their self-renewal capacity. This dysregulation in HSC function, characterized by hyperproliferation, partially elucidates the mechanism underlying CAR T-cell therapy-associated aplastic anemia [59, 60]. In addition, activated macrophages also contribute to this inflammatory milieu by secreting cytokines and chemokines, including ferritin, IL-1, IL-6, IL-10, TNF-α, and macrophage inflammatory protein-1 alpha (MIP-1α) [26, 57, 61]. MIP-1α impedes HSC proliferation, while TNF-α disrupts bone marrow hematopoiesis and erythrocyte integrity, exacerbating hematopoietic failure [60, 62] (Fig. 2).

#### Impaired HSCs and bone marrow microenvironment (BMM)

The HSC niche primarily comprises stromal cells and bioactive factors and is subject to modulation by the microenvironment [21]. The regenerative capacity of HSCs in patients undergoing CAR T-cell therapy is contingent upon a multitude of factors, including prior cytotoxic chemotherapy regimens, the natural ageing process, BMM and other variables. BMM plays a pivotal role in governing HSC maintenance, proliferation, and quiescence by secreting niche factors such as CXCL12 and stem cell factor (SCF) [63]. In a seminal investigation, Kitamura et al. identified that CD271^+^ niche cells are severely damaged in patients experiencing prolonged cytopenias. Furthermore, they observed a significant decrease in SCF essential for hematopoietic recovery in the bone marrow of patients with long-term cytopenias [63]. These findings shed light on a novel aspect of the pathophysiology underlying CAR T-cell therapy-associated cytopenias. Additionally, CAR T-cell therapy may have the potential to impede hematopoiesis in bone marrow by inducing an accumulation of reactive oxygen species (ROS) levels [64, 65]. It has been demonstrated that an excess of ROS production under conditions of oxidative stress can directly damage hematopoietic stem cells, resulting in impaired hematopoiesis [66, 67]. Moreover, an excess of ROS can result in a reduction of CAR T-cells, thereby impairing their ability to effectively combat tumors and potentially increasing the risk of hematological toxicity [68]. One of the current challenges in CAR-T cell research is the development of strategies to maintain the metabolic equilibrium of CAR-T cells in the bone marrow [69]. While there have been successful developments of CARs adapted to low-oxygen environments, they remain in the preclinical stage [70, 71]. It seems probable that further developments will be required before they can be applied clinically. Nevertheless, they may prove to be a significant breakthrough in addressing the aforementioned challenges.

#### Clonal hematopoiesis (CH) and secondary malignancy

CH and secondary primary malignancies (SPM), have recently garnered significant attention within the medical community [72–75]. CH encompasses a spectrum of precursor bone marrow malignancies, predominantly comprising clonal hematopoiesis of indeterminate potential (CHIP) and clonal hematopoiesis of unknown significance (CHUS) [76]. CHIP tends to be more prevalent in elderly patients, who may consequently exhibit heightened susceptibility to myelodysplastic syndrome (MDS) following CAR T-cell therapy attributable to the presence of CH [77]. Miller et al. observed the presence of CHIP in 48% of 154 patients with non-Hodgkin lymphoma (NHL) and MM undergoing CAR T-cell therapy. This was associated with an increased incidence of CRS severity [43]. The most frequent mutations were DNMT3A, TP53, TET2, PPMD1 and ASXL1, among which DNMT3A, ASXL1 and TET2 were closely related with inflammation. In contrast, Galli and colleagues reported no association between CHIP and CRS [78]. We speculate that CHIP may ultimately result in long-term cytopenias by promoting an inflammatory response. Long-term follow-up studies subsequent to CAR T-cell immunotherapy have indicated that SPM develops in approximately 15% of patients [75]. However, it remains unclear whether the emergence of secondary malignancies following CAR T-cell therapy is directly attributed to the CAR T-cells themselves. Further research endeavors are warranted to elucidate this aspect. We believe there is a possibility of correlating aberrant activation of CAR T-cells in vivo, but further research is needed to confirm this and gain a deeper understanding of the underlying mechanisms.

### Current prophylaxis and management of cytopenias

#### Early cytopenias

The incidence of cytopenias following CAR T-cell therapy is notably high (Table 2). Patients typically present with varying degrees of cytopenias within the initial 30 days post-infusion, with grade 3 to 4 neutropenia being the most prevalent [15, 53, 79]. Early supportive treatment with pure red blood cell (PRBC) or platelet transfusion is initiated when critical levels are reached to avert fatal bleeding and ameliorate symptoms of anemia.

Grade 4 neutropenia commonly emerges within the initial week and gradually ameliorates over a 10- to 14-day period following infusion, predisposing patients to a heightened risk of infections [20, 41]. Presently, the management of neutropenia associated with CAR T-cell therapy centers on supportive care, including the utilization of Granulocyte colony-stimulating factor (G-CSF) and anti-infective measures. The optimal timing for initiating G-CSF stimulation has been a topic of debate, owing to concerns regarding potential immunotoxicity (e.g., CRS and ICANS), compromised treatment response, and survival outcomes [80–82]. However, retrospective analyses have suggested an acceptable safety profile with early G-CSF administration, with no observed increase in the incidence of high-grade (≥ 3) CRS/ICANS [83, 84]. Lievin et al. have demonstrated that initiating G-CSF on the second day post-CAR T-cell infusion helps mitigate the risk of febrile neutropenia without exacerbating toxicity or impacting CAR T-cell kinetics [85]. In addition, EBMT and the EHA consensus do not recommend Granulocyte macrophage-colony stimulating factor (GM-CSF) for neutropenia post-CAR T-cell therapy [19, 86].

Concurrently, infection prophylaxis is imperative, with bacteria constituting the most common pathogens during early cytopenias, as evidenced in numerous retrospective analyses [87]. Jain et al. have advocated for the initiation of bacterial and fungal prophylaxis on day 0 or at the onset of first neutropenia, continued until absolute neutrophil count (ANC) recovery. Antiviral therapy is commenced promptly post-CAR T-cell therapy and sustained for 6 to 12 months, while anti-Pneumocystis jirovecii pneumonia (anti-PJP) prophylaxis is typically initiated on day 30 [13]. Moreover, adherence to the general EBMT and EHA consensus in 2023 suggests initiating anti-PJP and antiviral prophylaxis for all CAR T-cell therapy recipients, reserving antibacterial and antifungal prophylaxis for patients deemed high risk for severe (grade ≥ 3) ICAHT once ANC falls below 500/µL [19]. The optimal approach to managing cytopenias secondary to CAR T-cell therapy remains subject to ongoing investigation.

### Prolonged or late cytopenias

In addressing prolonged or late cytopenias post CAR T-cell therapy, bone marrow aspiration is a crucial diagnostic step to exclude primary disease progression/relapse or secondary malignancy [16, 88]. Following the exclusion of additional pathological processes, patients with an ANC below 500/µL may benefit from treatment with G-CSF to facilitate granulocyte recovery [9]. Moreover, initiation of anti-infective prophylaxis tailored to the patient’s individual risk profile for hematological cytopenias is imperative to mitigate the risk of infection [9, 19]. Patients experiencing severe anemia or thrombocytopenia post-CAR T-cell therapy necessitate transfusions as integral components of supportive care to avert fatal hemorrhage. Thrombopoietin receptor agonists (TPO-RAs) have emerged as promising agents for the treatment of prolonged thrombocytopenia, although consensus or standardization regarding their utilization across centers remains lacking [11, 29, 89, 90]. Erythropoietin-stimulating agents have also shown efficacy in managing persistent anemia.

Furthermore, anti-cytokine therapy (e.g., anakinra and tocilizumab) has been employed in certain institutions, notwithstanding uncertainty regarding their specific efficacy [40]. Sirolimus has been reported to reverse persistent grade 4 thrombocytopenia and severe platelet transfusion requirements in a case of DLBCL patient treated with CAR T-cells, without apparent compromise to CAR T-cell efficacy [91]. Additionally, Wang et al. have demonstrated that low-dose prednisone therapy can promote late hematologic recovery [92].

For patient refractory and/or unresponsive to G-CSF, a promising strategy entails the utilization of a hematopoietic stem cell boost (HSCB). This approach involves the infusion of cryopreserved autologous or allogeneic CD34^+^ hematopoietic cells obtained from a previous collection [93, 94]. A small case series has demonstrated the efficacy of HSCB in patients experiencing severe and prolonged cytopenias subsequent to CD19/B cell maturation antigen (BCMA) CAR T-cell therapies, leading to high engraftment rates, sustained neutrophil and platelet counts, and favorable survival outcomes [90, 93, 94] (Table 4). This phenomenon suggests, to some extent, that persistent cytopenias may stem from hematopoietic stem/progenitor cell dysfunction rather than solely immunological or microenvironmental factors [95]. The expert panel recommends that if autologous hematopoietic stem cells (AHSC) are available, HSCB without prior conditioning chemotherapy should be contemplated in patients experiencing ICAHT of grade ≥ 3 beyond day + 14 post-CAR T-cell infusion [96]. While several centers administer stem cell boosts to patients with severe cytopenias after CAR T-cell therapy, the optimal timing of such interventions remains to be determined [54, 93, 95]. The limited sample sizes, inaccurate optimal timing of treatment and narrow source of HSC currently impede the widespread application of HSCB.

In scenarios where the aforementioned therapeutic modalities prove ineffective for patients with severe profound cytopenias (grade ≥ 4), the expert panel from a single center recommends initiating allo-HSCT as salvage therapy. However, experience and evidence in this domain are presently limited [19]. Prior to embarking on allo-HSCT, meticulous evaluation of various variables is imperative, including the temporal proximity to CAR T-cell therapy, the likelihood of spontaneous recovery versus disease progression, the risk of fatal infection, donor suitability and availability, and the treatment objectives of the patient [97].

**Table 4:** Case series about the efficacy of HSCB for long-term cytopenias following CAR T-cell therapy

Source	*N*	Diagnosis *n* (%)	Type of product *n* (%)	Duration of neutropenia at the time of HSCB median (range)	CD34^+^progenitors (×10^6^/kg) BW, median (range)	ANC at time of boost (×10^9^/L), median (range)	Response rate (%)	OS
Yan, et al. [98]	1	R/R MM 1 (100)	CARTs-CD19/BCMA 1 (100)	35	3.05	<1	100	NA
Lipsitt, et al. [99]	1	R/R B-ALL 1 (100)	Tisagenlecleucel 1 (100)	69	5.1	NA (severe and prolonged cytopenias)	100	More than 3years
Rejeski, et al. [93]	12	R/R LBCL 9 (75) R/R ALL 2 (16) R/R MCL 1 (8)	Axicabtagene ciloleucel 9 (75) Tisagenlecleucel 2 (16) Brexucabtagene autoleucel 1 (8)	69 (35–617)	3.1 (1.7–7.5)	0.50 (0-7.9)	100	12-month OS 55%
Mullanfiroze, et al. [94]	7	R/R B-ALL 7 (100)	CARTs-CD19 5 (71) CARTs-CD19/CD22 2 (29)	78 (57–495)	6.75 (2.5–11.2)	grade 3 to 4 cytopenias	71	NA
Gagelmann, et al. [95]	31	R/R DLBCL 25 (81) R/R PMBCL 2 (7) R/R BL 1 (3) R/R cBALL 1 (3) R/R MCL 1 (3) R/R PCNSL 1 (3)	Axicabtagene ciloleucel 20 (64) Tisagenlecleucel 7 (23) Allogeneic 3 (10) Brexucabtagene autoleucel 1 (3)	38 (7-151)	3.6 (1.1–11.5)	0.2 (0–2.0)	84	12-month OS 74%
Mohan, et al. [100]	16	R/R MM	idecabtagene vicleucel 42 (39) ciltacabtagene autoleucel 14 (13) Investigational CARTs-BCMA 52 (48)	116 (29–270)	3.84 (1.05–9.04)	0.72 (0–2.6)	100	NA

Abbreviations HSCB: hematopoietic stem cell boost; R/R: relapsed/refractory; MM, multiple myeloma; DLBCL, diffuse large B cell lymphoma; B-ALL, acute B lymphoblastic leukemia; LBCL, large B cell lymphoma; ALL, acute lymphoblastic leukemia; MCL, mantle cell lymphoma; CAR-T, chimeric antigen receptor T-cell therapy; cBALL, common B acute lymphoblastic leukemia; BL, Burkitt lymphoma; PCNSL, primary central nervous system lymphoma; PMBCL, primary mediastinal B-cell lymphoma; OS, overall survival; BCMA, B cell maturation antigen; ANC, absolute neutrophil count; NA, not available; N means the total patients

### Prognosis

We conducted a comprehensive review of the published literature concerning HTs following CAR T-cell therapy, noting a paucity of reports on the overall survival (OS) and progression-free survival (PFS) outcomes among patients who develop prolonged hematological toxicity (PTH) subsequent to CAR T-cell therapy. Nagle et al. [29] have provided insights into this aspect, reporting that among 31 patients with R/R DLBCL treated with anti-CD19 CAR T-cell therapy, the 1-year OS rate in patients with PTH is notably lower compared to those without PTH (36% vs. 81%, *P* < 0.05). Similarly, Li et al. [17] have investigated 54 patients with R/R MM who undergo treatment with anti-BCMA/CD19 CAR T-cell therapy. Their findings reveal that the median PFS among PHT patients is 5 months, and the median OS is 24.5 months, significantly shorter than those observed in non-PTH patients. Furthermore, Liu et al. have observed that the 3-year OS rate among patients with R/R MM subjected to CAR T-cell therapy is markedly lower in the PTH group compared to the non-PTH group (41.5% vs. 66.5%, *P* < 0.001). Collectively, these studies underscore that the long-term prognosis of PTH patients tends to be inferior to that of non-PTH patients [17].

## CAR-T therapy-associated hemophagocytic lymphohistiocytosis (carHLH)

carHLH, known as IEC-HS, has been reported in approximately 2.7–14.8% of patients, representing a potentially life-threatening complication [27, 60, 101, 102]. carHLH manifests as an emerging clinical syndrome characterized by heightened activity of macrophages and lymphocytes, excessive secretion of pro-inflammatory cytokines, tissue infiltration, hemophagocytosis, and multi-organ dysfunction [23]. The onset of carHLH typically occurs around 10 to 14 days following CAR T-cell infusion. However, instances of late-onset carHLH, occurring after CRS resolution, have also been documented. Studies have revealed that the median time of death among these patients is approximately 44.5 days post-CAR T-cell infusion, with a mortality rate approaching 69.9% [60, 103]. These findings underscore the critical need for improved understanding, early recognition, and effective management strategies for carHLH to mitigate its devastating impact on patient outcomes.

### The diagnostic criteria of carHLH

Prior to the advent of CAR T-cell therapy, the HLH-2004 criteria and the H-score stood as the primary scoring systems utilized for diagnosing HLH [104]. However, several studies have indicated that these scoring systems may not be directly applicable to patients with carHLH due to the increased risk of false positives with the H-score and false negatives with the HLH-2004 criteria [105].

In response to these challenges, the CARTOX criteria are developed by the CARTOX Working Group in 2017 to facilitate the diagnosis of carHLH. According to these criteria, a diagnosis of carHLH is suggested if peak ferritin levels exceed 10,000 ng/mL during the CRS stage, accompanied by at least two of the following criteria: grade 3 or higher organ toxicity affecting the liver, kidney, lungs, or evidence of hemophagocytosis in the bone marrow or other organs [23]. Despite these efforts, distinguishing between severe CRS and CAR T-cell-related HLH/macrophage activation syndrome (MAS) remains a challenge [106]. More recently, the ASTCT has developed new diagnostic criteria in an effort to differentiate IEC-HS from severe CRS and ICANS [57]. A comparison of these diagnostic criteria is detailed in Table 5. These evolving diagnostic frameworks aim to differentiate it from other CAR T-cell therapy-associated toxicities, thereby facilitating prompt and targeted management strategies.

In addition, cytokines such as IL-1, IL-6, IFN-γ, and TNF-α play a significant role in the pathophysiological pathogenesis of carHLH. However, they were not previously included in the diagnostic criteria due to the concern that intersections with the cytokine profiles of CRS might lead to an inaccurate diagnosis. In a recent study, Huang and colleagues demonstrated that a distinctive cytokine network, characterized by elevated levels of IFN-γ, granzyme B, IL-1RA and IL-10, can effectively differentiate carHLH from CRS. They also developed a predictive model for carHLH. In light of these findings, it seems prudent to suggest that improvements could be made to the specificity and sensitivity of the current diagnostic criteria for carHLH. In the future, the diagnostic efficacy may be enhanced by incorporating cytokine networks, thereby assisting clinicians in making prompt and accurate diagnoses and implementing timely interventions to improve patient survival and prognosis. It is, however, necessary to confirm this in future clinical studies with larger cohort.

**Table 5:** Comparison of diagnostic criteria of CAR T-cell therapy associated HLH

HLH-2004 criteria [107]	CARTOX criteria [23]	ASTCT criteria [57]
**Meet at least 5 of the following criteria** • Fever ≥ 38.5 °C • Splenomegaly • Cytopenias (more than two lineages) Hemoglobin < 90 g/dL Platelets < 100 × 10^9^/L ANC < 1.0 × 10^9^/L • Hypertriglyceridemia>265 mg/dL and/or hypofibrinogenemia < 1.50 g/L • Hemophagocytosis in bone marrow, spleen, or lymph node • Ferritin > 500 µg/L • Soluble CD25 (i.e., soluble IL-2 receptor) ≥ 2,400U/ml • Low/absent NK-cell-activity	• Ferritin levels peak at > 10,000 ng/mL during the CRS phase of CAR-T therpay • **Any two of the following**: a. Grade ≥ 3 increase in serum bilirubin, aspartate aminotransferase, or alanine aminotransferase levels b. Grade ≥ 3 oliguria or increase in serum creatinine levels c. Grade ≥ 3 pulmonary edema d. Presence of hemophagocytosis in bone marrow or organs	• **Most common manifestations** a. Required: elevated ferritin (> 2×ULN or baseline and/or rapidly rising) b. Onset with resolving/resolved CRS or worsening inflammatory response after initial improvement with CRS-directed therapy c. Hepatic transaminase elevation (> 5×ULN or > 5×baseline) d. Hypofibrinogenemia (< 150 mg/dL or < LLN) e. Hemophagocytosis in bone marrow or other tissue f. Cytopenias • **Other manifestations that may be present** a. Lactate dehydrogenase elevations (> ULN) b. Other coagulation abnormalities (e.g., elevated PT/APTT) c. Direct hyperbilirubinemia d. New-onset splenomegaly e. Fever (new or persistent) f. Neurotoxicity g. Pulmonary manifestations (e.g., hypoxia, pulmonary infiltrates, pulmonary edema) h. Renal insufficiency (new onset) i. Hypertriglyceridemia (fasting level, > 265 mg/dL)

Abbreviations HLH, hemophagocytic lymphohistiocytosis; ANC, absolute neutrophil count; ULN, upper limit of normal; NK-cell-activity, Natural Killer-cell-activity; LLN, lower limit of normal; PT, prothrombin time; APTT, activated partial thromboplastin time; IL, interleukin.

### Risk factors for carHLH

Pre-existing severe CRS emerges as the most significant risk factor for carHLH. Numerous studies have underscored its pivotal role in triggering HLH development [60]. Additionally, several other factors have been implicated, including high disease burden, elevated ferritin levels prior to carHLH onset (> 20,000 ng/mL in 24 h), prolonged CRS duration, duration of CAR T-cell expansion, and prior infections [103, 108]. Notably, carHLH may also exhibit an association with the distinct costimulatory domains of CAR T-cell products, specifically CD28 and 4-1BB for axicabtagene and tisagenlecleucel, respectively [60]. ZUMA-1 and JULIET trials have indicated differences in carHLH incidence between CAR T-cell products. For instance, in ZUMA-1, one out of 119 patients treated with axicabtagene succumb to HLH, whereas no HLH-related deaths are reported among 167 patients treated with tisagenlecleucel [24, 25]. Shah et al. have observed a higher incidence of HLH, approximately 31.8%, in patients administered with CD22 CAR T-cells [109]. These findings highlight the importance of considering specific CAR T-cell products and their associated risk profiles in assessing the likelihood of carHLH development. Here, we present the initial systematic and comprehensive summary of the risk factors associated with carHLH (Table 6).

**Table 6:** Risk factors related to carHLH

Risk factors
higher CRS grade [60, 110]
peak serum CAR concentration [103]
higher maximum IFN-γ, CXCL9, CXCL10, granzyme B, TNFα, IL-6, IL-10, IL-1βand IL-18 [110–112]
high disease burden [103, 108]
higher baseline CRP, ferritin [103]
Increased T: NK ratio prior CAR-T therapy [113]
duration of CRS, CAR T-cells [108, 114]

Abbreviations CRS, cytokine release syndrome; CAR, chimeric antigen receptor; IFN-γ, interferon γ; CXCL, CXC chemokine ligand; TNFα, tumor necrosis factor-alpha; IL, interleukin; CRP, C-reactive protein; T: NK ratio, T cell: Natural killer cell ratio

### Mechanisms of carHLH

**Fig. 3:**
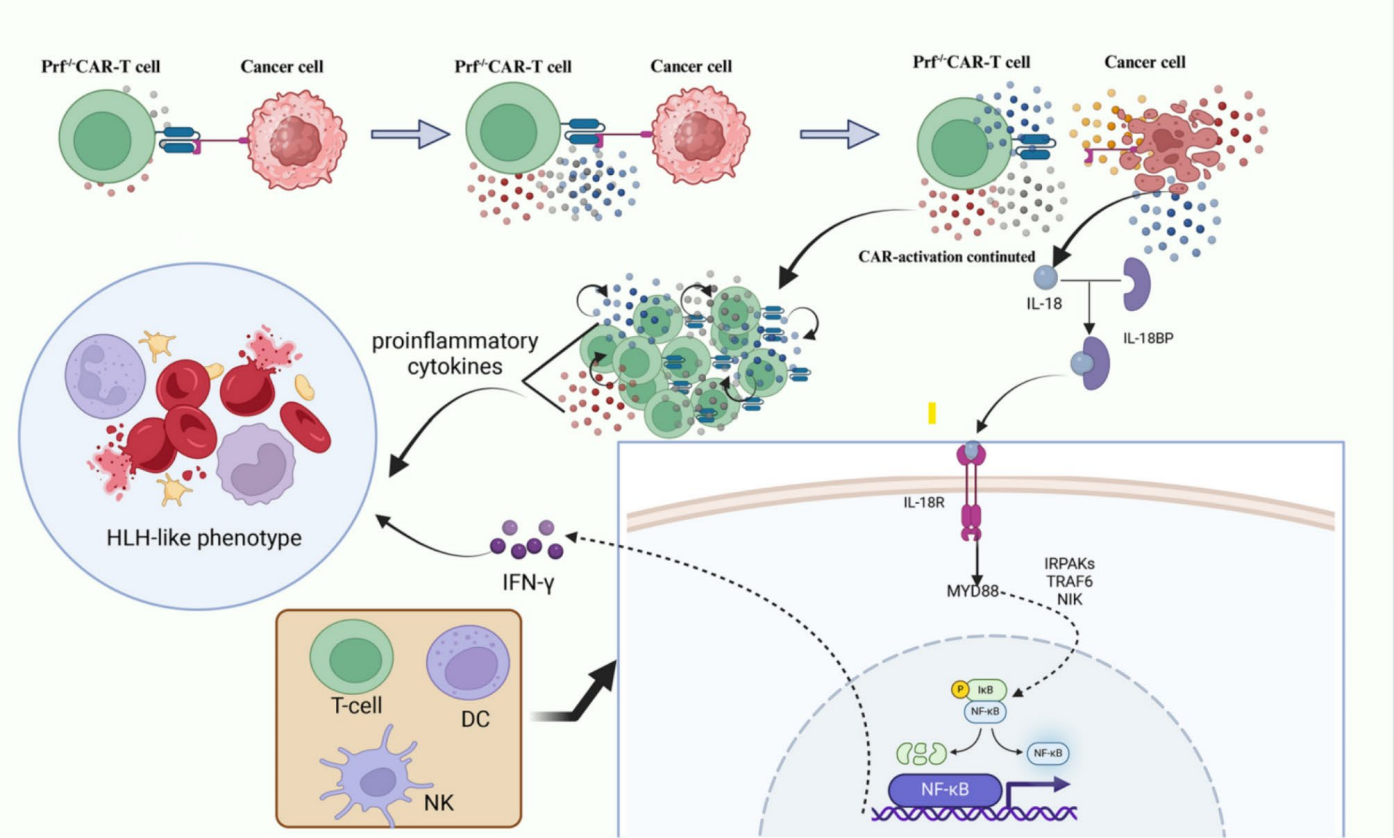
The potential mechanism of carHLH. The activation of CAR T-cells leads to the release of significant quantities of cytokines, including IFN-γ, TNF-α, IL-6, IL-10, and GM-CSF. In the absence of perforin, CAR T-cells undergo persistent activation and expansion, subsequently generating and releasing copious amounts of inflammatory cytokines and pro-inflammatory mediators. Additionally, the continuous targeting and destruction of tumor cells by CAR T-cells result in the lysis of these cells, leading to the release of cytokines, such as IL-18. Under normal circumstances, free IL-18 binds to IL-18BP, forming high-affinity complexes that serve to mitigate any deleterious systemic effects of IL-18. However, when the release of IL-18 surpasses the regulatory capacity of IL-18BP, free IL-18 can engage with the IL-18 receptor, predominantly expressed on T cells, NK cells, dendritic cells, and other IFN-γ-secreting cells. This binding initiates a signaling cascade that activates the NF-κB pathway, consequently leading to the synthesis and release of significant quantities of IFN-γ. IFN-γ, in turn, activates macrophages, resulting in the secretion of elevated levels of inflammatory cytokines, thereby contributing to the development of delayed carHLH. Abbreviation: prf^−/−^: perforin-deficient CAR-T cells; IL, interleukin; DC, dendritic cell; NK, natural killer cell; IFN-γ, interferon-γ; IL-18BP, IL-18 binding protein; IL-18R, IL-18 receptor; NF-κB, Nuclear factor kappa B

A number of factors have been identified as potentially involved in the etiology of carHLH. The activated CAR T-cells have been found to liberate substantial quantities of cytokines, including IFN-γ, TNF-α, IL-6, IL-10, and GM-CSF. These cytokines may prompt inflammatory responses [48, 109]. Furthermore, the absence of perforin, a phenomenon associated with primary HLH, has recently been linked to the cytotoxic activity of CD8^+^ and CD4^+^ CAR T-cells [115]. In the absence of perforin, CAR T-cells may undergo re-expansion and subsequently produce heightened levels of proinflammatory cytokines, such as GM-CSF, TNF-α, and IFN-γ, which ultimately result in the initiation of an inflammatory cascade reaction and the subsequent onset of delayed HLH following CRS resolution [115] (Fig. 3).

Additionally, tumor cell pyroptosis leads to the release of a plethora of inflammatory and pro-inflammatory cytokines, notably IL-18 [10]. Ordinarily, free IL-18 complexes with IL-18 binding protein (IL-18BP) forming high-affinity complexes that mitigate any deleterious systemic effects of IL-18 [116, 117]. Recent investigations have evinced a significant elevation in free IL-18 levels among patients afflicted with CD22-associated carHLH relative to those experiencing CRS alone [112]. Excessive IL-18 releases, surpassing the regulatory capacity of IL-18BP, permits free IL-18 to engage the IL-18 receptor, predominantly expressed on T cells, natural killer cells, dendritic cells, and other IFN-γ-secreting cells. This interaction initiates signaling cascades activating nuclear factor kappa B (NF-κB) pathways or STAT3, MAPK, and JNK pathways, culminating in the robust generation and dissemination of IFN-γ [117, 118]. Moreover, patients with carHLH exhibit significantly elevated serum ferritin levels, which can potentiate the release of inflammatory cytokines by activating the NF-κB pathway [119]. IFN-γ activation of macrophages may induce the release of elevated levels of inflammatory cytokines (IL-1β, IL-6, IL-12, and TNF-α) and the inflammatory chemokine MIP-1α, eventually contributing to cytokine storm. Up to date, the precise correlation and underlying mechanisms necessitate further inquiry (Fig. 3).

### Management of carHLH

Presently, a paucity of standardized and efficacious methodologies exists for the clinical management of carHLH, with most therapeutic approaches grounded in expert consensus. Following CAR T-cell infusion, vigilant monitoring of patient’s vital signs, hematological parameters, hepatic and renal functions, serum ferritin, and triglyceride levels is imperative to promptly discern any alterations in their clinical status [9, 104]. Subsequently, systemic corticosteroids either alone or in conjunction with other anti-inflammatory agents constitute the cornerstone of treatment protocols for carHLH, both initially and subsequently, prior to the escalation of life-threatening symptoms [23, 57, 104]. Following guidelines for CRS and recommendations from the National Comprehensive Cancer Network (NCCN) clinical practice guidelines, patients suspected of carHLH should receive anti-IL-6 therapy and corticosteroids for grade ≥ 3 organ toxicity [23, 120]. In individuals lacking severe end-organ damage, concomitant neurotoxicity, or refractory grade 4 hypotension, an initial dose of intravenous dexamethasone 10 mg every 6 h is advised, with escalation to 20 mg intravenously every 6 h in refractory cases, coupled with a rapid tapering regimen [23]. Moreover, the ASTCT IEC-HS Committee advocates for the early initiation of IL-1 receptor antagonists (e.g., anakinra) upon the onset of carHLH [57]. Conversely, for carHLH patients exhibiting end-organ toxicity, “pulse doses” of dexamethasone ranging from 5 to 10 mg/m^2^ every 6–24 h are recommended [121]. If clinical or serological improvement not manifest within 48 h, consideration should be given to administering etoposide at doses of 75–100 mg/m^2^ [121, 122]. Additionally, ASTCT guidelines suggest commencing JAK1/2 inhibitors (e.g., ruxolitinib) alongside corticosteroids and augmenting the dosage of anakinra IL-1 blocker as a secondary therapeutic option for IEC-HS [57]. In instances where a patient’s condition demonstrates continued deterioration, IFN-γ antagonists such as epalutumab have exhibited promising efficacy in managing refractory and progressive IEC-HS in select case series and trials [123, 124]. Nevertheless, the imperative for further prospective clinical trials persists to comprehensively elucidate the management paradigms for carHLH in the foreseeable future.

## CAR-T-associated coagulopathy (CARAC)

Coagulopathy, also known as CARAC, represents one of the most prevalent HTs associated with CAR T-cell therapy, exhibiting an incidence ranging from 51 to 56.6%. Typically manifesting within 28 days following CAR T-cell infusion, the majority of occurrences arise between 6 and 10 days post-infusion [119, 125]. Notably, approximately 19.6% of patients afflicted with CARAC experience clinically significant bleeding, while 14–50% subsequently progress to develop disseminated intravascular coagulation (DIC), with mortality rates ranging from 6.7 to 42.9% among those diagnosed with DIC [126].

### Risk factors for CARAC

Patients presenting with CARAC typically demonstrate a spectrum of abnormal coagulation parameters, primarily characterized by prolonged activated partial thromboplastin time (APTT) and prothrombin time (PT), reduced fibrinogen levels, and elevated D-dimer and fibrin degradation product levels [15, 126, 127]. Furthermore, the severity of CARAC has been observed to positively correlate with the grade of CRS; specifically, patients experiencing CRS of grade ≥ 3 tend to manifest more pronounced coagulopathy [125, 128]. Risk factors associated with the development of CARAC include the rapid expansion of CAR T-cells, high-grade CRS, severe neurologic toxicity, and elevated pre-infusion tumor burden [9, 128, 129]. Additionally, a low baseline platelet counts and the occurrence of high-grade ICANS have been identified as predisposing factors for bleeding complications subsequent to CAR T-cell therapy.

### Mechanisms of CARAC

To date, numerous studies have endeavored to elucidate the underlying causes of CARAC [129]. Notably, high-grade CRS, severe ICANS, and the lymphocyte count at day 0 have been independently identified as factors associated with CARAC. Zhang et al. have proposed that the activation of platelets, monocytes, endothelial cells, and the ensuing CD40/CD40L interactions between them collectively contribute to CRS-related coagulopathy [122]. In individuals experiencing severe CRS, elevated levels of cytokines such as IL-6 and TNF-α induce hepatic insufficiency, impairing the synthesis of coagulation factors [130]. Simultaneously, elevated cytokine levels activate and damage endothelial cells, leading to the exposure of subendothelial collagen fibers and subsequent activation of both endogenous and exogenous coagulation pathways. Additionally, the off-target effects of CAR T-cells can incite hepatocyte damage, further compromising the synthesis of coagulation factors.

Recent findings from a study employing proteomic analysis have identified 10 differentially expressed proteins primarily involved in immune response, complement and coagulation pathways, and fibrinolysis pathways. These proteins include complement3 (C3), C4a, C4b, mannose-binding lectin 2 (MBL2), C8A, C8b, CRP, alpha-2-macroglobulin (A2M), clusterin (CLU), and fibrinogen alpha chain (FGA) [131]. This evidence underscores the significant roles of complement activation and the acute inflammatory response in the pathogenesis of CARAC. However, despite these insights, the precise molecular mechanisms underlying CARAC necessitate further elucidation [131].

### Management of CARAC

Patients undergoing CAR T-cell therapy commonly exhibit abnormal coagulation parameters and severe CRS, often classified as grade 3 or higher. Buechner et al. have proposed that in individuals with CRS-related coagulopathy and concurrent severe hypofibrinogenemia, initiation of replacement therapy with fibrinogen concentrates is warranted when the fibrinogen level falls below 1.5 g/L. This intervention should be promptly assessed, with fibrinogen levels measured 30 to 60 min after the first replacement infusion [132]. If prothrombin time (PT) is prolonged ≥ 3 s and/or activated partial thromboplastin time (APTT) prolongation is ≥ 10 s, fresh frozen plasma may be infused if needed [126, 133]. Concurrently, corticosteroids and tocilizumab emerge as effective modalities for CRS management, exerting their therapeutic effects by mitigating the levels of various cytokines. This consequential reduction in cytokine levels can subsequently ameliorate coagulopathy and DIC [125, 126].

## B-cell aplasia

The unique toxicity profile associated with CAR T-cell therapy, termed the on-target off-tumor effect, arises from the direct assault on normal tissues [134]. Given that CD19-directed CAR T-cells target normal B cells, adverse effects such as B-cell aplasia and hypogammaglobulinemia are anticipated subsequent to anti-CD19 or anti-CD22 CAR T-cell therapy, potentially predisposing individuals to various infectious diseases [135, 136]. B-cell aplasia stands as a hallmark of CAR T-cell persistence and efficacy, also serving as a predictive indicator for disease relapse. Notably, the premature resolution of B-cell aplasia following CD19-directed CAR T-cell infusion frequently correlates with an augmented risk of relapse [136, 137]. However, contrary findings suggest that despite an ongoing response to CAR T-cell therapy, B cells within the lymphoma milieu may exhibit recovery [24, 88, 138].

In terms of risk factors, recent investigations have identified low baseline hematopoietic reserves and subsequent inadequate immune reconstitution across multiple lineages, including B cells, T cells, and myeloid cells, as pivotal contributors to the development of B-cell aplasia [139]. Additionally, the composition of CAR T-cell constructs has been linked to the duration of B-cell aplasia, with B-cell recovery occurring within 1–2 months with CD28-based CAR T-cells, and later with 41BB-based CARs [15].

The resultant hypogammaglobulinemia stemming from B-cell aplasia may elevate the risk of infection. While short-term B-cell aplasia may not necessitate treatment, prolonged instances may warrant immunoglobulin replacement therapy, particularly in pediatric populations [140]. Current guidelines advocate for the consideration of intravenous immunoglobulin in cases of severe or recurrent infections [141].

## Conclusions

In summary, the clinical presentation of patients with HTs following CAR T-cell therapy is diverse, often posing challenges in diagnosis and potentially leading to misdiagnosis or underdiagnosis, particularly due to the overlapping symptoms. The underlying mechanisms of HTs remain incompletely understood, and there exists no consensus regarding their management across different countries. This lack of consensus may pose risks to patient safety, compromise their quality of life, prolong hospitalization durations, and escalate the burden on medical resources significantly. Addressing these challenges necessitates a dual focus on elucidating the pathogenesis of HTs and optimizing the therapeutic efficacy of CAR T-cell therapy while mitigating the risk of HT occurrence. Achieving this goal represents a formidable task for the future. Furthermore, concerted efforts to conduct prospective clinical studies and delve into the molecular pathways implicated in HTs hold promise for enhancing the treatment outcomes of CAR T-cell therapy in clinical settings.

## Data Availability

No datasets were generated or analysed during the current study.

## Abbreviations

CAR: Chimeric antigen receptor
CRS: Cytokine release syndrome
ICANS: Immune effector cell-associated neurotoxicity syndrome
HTs: Hematological toxicities
HLH: Hemophagocytic lymphohistiocytosis
R/R: Relapsed/refractory
FDA: Food and Drug Administration
LBCL: Large B cell lymphoma
DLBCL: Diffuse Large B-cell lymphoma
MCL: Mantle cell lymphoma
FL: Follicular lymphoma
BCMA: B cell maturation antigen
MM: Multiple myeloma
CLL/SLL: Chronic lymphocytic leukemia/small lymphocytic lymphoma
ICAHT: Immune effector cell-associated hematological toxicity
EHA: European Hematology Association
EBMT: European Society for Blood and Marrow Transplantation
ECOG: Eastern Cooperative Oncology Group
B-ALL: B cell acute lymphoblastic leukemia
allo-HSCT: Allogeneic hematopoietic stem cell transplantation
LD: Lymphodepletion
FC: Fludarabine/cyclophosphamide
IL: Interleukin
SDF-1: Serum stromal cell-derived factor-1
CXCL: C-X-C motif chemokine ligand
HSC: Hematopoietic stem cell
IFN: Interferon
TNF: Tumor necrosis factor
TGF-β1: Transforming growth factor-β1
ScFv: Single-chain Fv
CHIP: Clonal hematopoiesis of indeterminate potential
MDS: Myelodysplastic syndrome
ANC: Absolute neutrophil count
GM-CSF: Granulocyte macrophage-colony stimulating factor
MIP-1α: Macrophage inflammatory protein-1 alpha
BMM: Bone marrow microenvironment
SCF: Stem cell factor
HSPCs: Hematopoietic stem and progenitor cells
CH: Clonal hematopoiesis
SPM: Secondary primary malignancy
DLBCL: Diffuse large B cell lymphoma
NK: cell Natural killer cell
PRBC: Pure red blood cell
G-CSF: Granulocyte colony-stimulating factor
anti-PJP: Anti-Pneumocystis jirovecii pneumonia
TPO-Ras: Thrombopoietin receptor agonists
HSCB: Hematopoietic stem cell boost
AHSC: Autologous hematopoietic stem cells
PHT: Prolonged hematological toxicity
OS: Overall survival
PFS: Progression-free survival
ASTCT: American Society for Transplantation and Cell Therapy
carHLH: CAR T-cell therapy-associated hemophagocytic lymphohistiocytosis
IEC-HS: Immune effector cell-associated HLH-like syndrome
NF-κB: Nuclear factor kappa B
IL-18BP: IL-18 binding protein
MAS: Macrophage activation syndrome
NCCN: National Comprehensive Cancer Network
CARAC: CAR-T-associated coagulopathy
DIC: Disseminated intravascular coagulation
MBL2: Mannose-binding lectin 2
CLU: Clusterin
FGA: Fibrinogen alpha chain
PT: Prothrombin time
APTT: Activated partial thromboplastin time
ROS: Reactive oxygen species
